# P-11 - INVASIVE GROUP A STREPTOCOCCAL INFECTION OUTBREAKS AND CLUSTERS ASSOCIATED WITH OUTPATIENT AND AMBULATORY CARE SETTINGS IN SOUTH-EAST ENGLAND 2015-2025: DESCRIPTIVE STUDY AND NARRATIVE LITERATURE REVIEW

**DOI:** 10.1093/pubmed/fdag046.080

**Published:** 2026-07-09

**Authors:** Dr Rebecca Bestwick, Dr Samuel Healy, Dr David J Roberts

**Affiliations:** UK Health Security Agency, South East; UK Health Security Agency, South East; UK Health Security Agency, South East

## Abstract

**Background:**

Invasive Group A streptococcus (iGAS) is associated with substantial morbidity and mortality. The incidence of reported outbreaks is increasing, and wound care settings are recognised risk environments. Outbreaks have been described in acute and maternity settings and home-based care, however, there are limited reports from other settings with potentially similar risk exposures - such as primary care, outpatient and ambulatory care (OAC) settings. A potential under-recognition in OAC settings may result in suboptimal responses following iGAS cases.

**Objectives:**

To describe outbreaks and clusters of iGAS infection in OAC settings in the South East region (SE), alongside a focused review of the published literature, to inform surveillance and response actions.

**Methods:**

Outbreaks/clusters were identified through a structured query of the Health Protection Team (HPT) record management system. We extracted detail from those that met pre-set inclusion criteria and presented the findings using descriptive frequency statistics. iGAS cases and outbreaks were defined aligning with public health guidance and adapted for the OAC setting. For the narrative review, we searched PubMed for outbreak reports published between 2000 and 2025.

**Results:**

Within the SE, seven OAC outbreaks and no clusters were identified. All outbreaks occurred in wound care settings (four in outpatient clinics, two in primary care, and one in a podiatry clinic). Cases were typically older patients presenting with severe cellulitis. SE outbreaks were comparatively smaller with fewer identified IPC lapses compared to those reported in the literature; only three reports were identified from 1535 screened publications.

**Conclusions:**

Previous awareness of the risk of iGAS outbreaks in the context of wound care in OAC settings may have been limited. For HPTs, our findings illustrate importance of careful risk assessment and outbreak surveillance even after a single case

